# A Framework for a European Economic Recovery After COVID-19

**DOI:** 10.1007/s10272-020-0904-2

**Published:** 2020-07-28

**Authors:** Julia Anderson, Simone Tagliapietra, Guntram B. Wolff

**Affiliations:** Bruegel, Rue de la Charité 33, 1210 Brussels, Belgium

## Abstract

The success of support measures as COVID-19 lockdowns are relaxed depends on the type of recovery the EU wants to achieve.

To slow the spread of COVID-19, European governments have adopted stringent containment measures. These have led to a severe recession, and policymakers in European Union countries are providing ample support to help companies cope with the immediate consequences. The basic approach has been to provide generous and indiscriminate emergency support to help cash-strapped firms meet their immediate liquidity needs. But even as economies tentatively reopen, countries face deep recessions and a more comprehensive strategy for the future needs to be designed.

**Julia Anderson**, Bruegel, Brussels, Belgium.

**Guntram B. Wolff**, Bruegel, Brussels, Belgium.

The success of support measures as COVID-19 lockdowns are relaxed depends on the type of recovery the EU wants to achieve. At the same time, decisions taken today will have long-term implications for the single market and government debt. How should further fiscal support provided to companies be structured? What implications will different approaches have for the single market, government budgets and the EU's climate strategy? Difficult trade-offs lie ahead: a speedier recovery could run counter to green ambitions; national rescues could hurt neighbouring markets. Hard choices in the coming phases should follow a set of four principles and the recovery effort should be structured around equity and recovery funds with borrowing at the EU level.

## Three phases of economic response to COVID-19

COVID-19 lockdown measures have led to sharp contractions in economic output, household spending, corporate investment and international trade. EU countries have seen an estimated average decline in annual GDP growth of up to three percentage points per month of lockdown.[1]

1 Each month of lockdown is expected to cause a decline in annual GDP growth of 2.4 percentage points in Germany and of three percentage points in France and Italy.

The EU economy is predicted to contract by a record 7.4% in 2020 (European Commission, 2020a).

The impact of COVID-19 on the European economy might ultimately turn out to be even greater than currently estimated. The health and economic impact of the pandemic and the containment measures on sectors and countries have varied significantly. For example, tourism slowdowns have hit airlines and Mediterranean countries particularly hard. The construction sector is more heavily affected in some countries than in others (with construction production growth rates ranging from -61% in France to 0.7 in the Netherlands).[2]

In the fiscal economic policy response to the pandemic, three phases can be broadly distinguished.

Phase 1 measures are meant to temporarily freeze economies at the point they were at before the crisis in order to shield healthy businesses from bankruptcy and to protect European firms from hostile takeovers by foreign state-backed enterprises. Phase 1 support has been crude and indiscriminate, and rightfully so. The motto is speed over perfection. The national economic measures taken in phase 1 are characterised by indiscriminate, national liquidity support to firms and workers. These measures are meant to keep firms afloat in the face of near-universal cash shortfalls, to prevent unnecessary layoffs and to deter hostile takeovers (especially from non-EU state-financed enterprises).

As early as 19 March 2020, the European Commission amended the EU state aid rules with a so-called Temporary Framework to allow governments to undertake such measures. However, the size of fiscal responses in different EU countries has varied widely. For instance, immediate fiscal stimuli have ranged from 3% of GDP in Italy and 2% in Spain to 12% of GDP in Germany (Anderson et al., 2020).

Phase 2 is about solvency support. After months of lockdown, firms must take on increasing amounts of debt and draw on equity reserves to meet their working capital and investment needs. The European Commission estimates that by the end of 2020, 25% to 35% of European companies will have exhausted their working capital and liquidity buffers, falling short of an estimated €350 billion to €500 billion in liquidity (European Commission, 2020b).[3] At the same time, credit standards are tightening.[4] For increasingly leveraged firms, bankruptcy looms. Solvency support through direct recapitalisation is needed. This phase is expected to last roughly until the end of any lockdown measures. Lockdowns may continue until full immunity or a vaccine is available, so possibly well into 2021.

Phase 3 will then be about recovering from the severe contraction phase that the likely on-and-off switching of lockdown measures will leave in its wake. On 21 July 2020, the European Council agreed on an EU recovery fund, called Next Generation EU, targeting the sectors and geographical parts of the EU most affected by the crisis. However, only a very small share of the planned €750 billion will be spent in 2020.[5]

In the following, we discuss the key principles that should guide support measures in phases 2 and 3. One key consideration is that decisions taken in phase 2 – who gets bailed out, how and under what conditions – will determine who is left standing in the recovery phase. Conversely, predictions about the shape of the recovery and about policy measures enacted in phase 3 (such as demand support) could determine whether or not a company can be deemed solvent today.

As countries move to the next phases, taking account of EU cross-border effects will become increasingly important. Phases 1 and 2 have so far largely involved national fiscal policy. However, differences in state-aid disbursements and other support during phase 2 could well leave lasting marks as countries make different and uncoordinated decisions, whether due to fiscal space or preferences. Decisions taken now will thus shape the single market of tomorrow. In phase 3, economic outcomes will be shaped by budget decisions related to the EU's multiannual financial framework (MFF) and the EU recovery fund, alongside national recovery programmes. A comprehensive strategy for phases 2 and 3 is needed.

## Four guiding principles for managing phases 2 and 3

Moving from phase 1 to the next phases is not simply a matter of providing equity instead of debt. While phase 1 injections have been emergency measures, phase 2

2 Total construction, April in respect to February 2020. Eurostat data: https://ec.europa.eu/eurostat/statistics-explained/index.php?title=Impact_of_Covid-19_crisis_on_construction#Development_by_country.

3 Baseline scenario.

4 As reported in the 2020 Q1 European Central Bank bank-lending survey.

5 While the European Commission's 27 May 2020 proposal included an increase in the 2020 EU budget (€11.5 billion), the Council's agreement does not. However, some measures taken since the crisis "should be eligible for financing under [the] ReactEU and Recovery and Resilience Facility [programmes], provided they pursue objectives of the respective programmes" (European Council, 2020).

requires a long-term plan. It also requires recognition of difficult trade-offs ahead: speedy economic recovery versus environmental goals, health of the private sector versus public indebtedness, solvency versus social cohesion.

The job now for policymakers is to make clear the principles guiding their recovery strategies. Such principles should consistently inform policymakers' choices between the possible measures and the inevitable trade-offs. Can they ensure that rescue plans designed today do not cause unintended damage tomorrow?

But before reflecting on the principles that should guide future economic support, it is worth highlighting why such support is warranted in the first place. First, governments impose lockdown measures to achieve a public good: a healthy population. It is therefore appropriate that the public contributes to paying for the economic fallout from achieving that public good. Second, without further support, many jobs will be lost. Third, with numerous companies failing, invaluable tangible and intangible capital will be destroyed. Rebuilding that capital and founding new firms will take many years, during which time human capital will be permanently destroyed.

However, governments cannot and should not rescue every company with unlimited amounts of cash. This would be fiscally irresponsible and could cost the single market. A careful balance must be struck between public welfare objectives and the social, economic and political risks of rescue programmes.

We consider four principles to be of the utmost importance in this evaluation.

*First, only financially viable firms should receive solvency support, with financial viability assessed in terms of both the past and future*. Taxpayers should not support firms that were in bad shape before the virus-induced lockdowns, but assessments of financial viability need to go beyond published 2019 financial accounts.

The crisis may well alter consumer preferences and production systems. Public resources must focus on firms with business models that are expected to be viable in the post-crisis economy. Rescue plans should not be about preserving pre-crisis industrial structures. The recovery should be about jump-starting a healthy post-COVID-19 economy, which could mean letting some firms fail. Meanwhile, a forward-looking approach suggests financing the promising start-ups of the post-crisis economy. A key question here is who should conduct these forward-looking assessments?

We favour a mechanism in which the expertise of private investors is used to support decisions on the allocation of rescue funds. Such a system would be more transparent and accountable than if politicians and their administrations are left to decide unilaterally which companies to help. Involving private investors would help ensure that investments are viable in the long run, especially if they have a direct interest. Even so, credit tightening might lead to under-investment and the public sector therefore plays an important role.

The local knowledge and analytical capabilities of commercial banks are already extensively used to distribute state guarantees and subsidised loans to firms and individuals. Further partnerships will be required for equity-based instruments, especially for the more arduous assessments of the viability of smaller companies.

*Second, state support should not undermine competition in the EU's single market*. One of the EU's main strengths is well-functioning competition within its single market. Fair competition across borders ensures that the most innovative and productive firms thrive, rather than those that receive the most state support.

Relaxed state-aid rules allow EU governments to inject liquidity into cash-deprived registers. Inevitably, some countries will provide more generous support than others (Germany accounts for approximately half of the COVID-19 state aid approved by the Commission as of 1 May 2020). These differences risk distorting competition, especially if they continue during phase 2. At the extreme, fears of competitive disadvantage could trigger subsidy wars between EU countries, leading to huge wastes of public money (Motta and Peitz, 2020). The longer these differences persist, the more the single market and therefore the foundation of Europe's long-term growth will be affected.

Quantitative limits on the amounts of aid (e.g. the €800,000 cap on grants) impose some discipline (Neven, 2020). Nevertheless, some countries will deliver less than the maximum authorised amounts, while others will go beyond, taking advantage of the fact that aid provided under the Temporary Framework can be cumulated with other types of state aid.[6] Furthermore, quantitative limits on aid to individual firms do not prevent major differences in the scope of deployment. Indeed, firms that operate in economically less-affected countries will be at a great advantage compared to firms that deal with insolvent suppliers and clients in their daily business.

6 For example, €200,000 of de minimis aid and aid under Article 107(2) (b), which permits governments to compensate firms for incurred direct damage.

Rules to restrain the behaviour of artificially competitive firms also work to limit further distortions of the single market. To that end, the Commission's state-aid amendments prohibit aid-infused firms from engaging in aggressive commercial expansions and from acquiring rivals while they are repaying the state. These rules are welcome additions to the Commission's arsenal. However, these rules rely on vague behavioural notions that are not easy to enforce – when is a pricing strategy 'aggressive' and when is it procompetitive? – and are distortionary in their own right.[7]

*Third, state aid should support and not undermine the achievement of broader societal goals*. The EU and its members have set themselves societal goals including climate neutrality and social cohesion. It would be absurd if public funds now subsidised the business models that need fundamental change.

As governments engage in bilateral negotiations with firms, they are in a uniquely strong position to push for the changes that normally require years of regulating to implement. Support given to firms should be conditional on making the changes required to achieve the EU's societal objectives.

Putting conditions on state aid will require that difficult technical questions be addressed – around monitoring and enforcement, for example. Political disagreements, e.g. over conditions on dividends and bonuses attached to equity injections or environmental obligations, will have to be resolved. Indeed, a clear definition of broader societal goals needs to be agreed and supported by the entire EU. If the goals in different countries diverge too much, there will be a risk of further market distortions, with some firms held to much higher standards (for example, on environmental protection) than others. In light of these difficulties, and under pressure to act fast, it will be tempting to postpone these discussions until after the crisis.[8] But this would be a rare opportunity missed.

*Fourth, taxpayers should receive their share of the rewards of the recovery*. Generous support schemes funded by the taxpayer should give the taxpayer some claims on future profits. Moving beyond emergency rescues, interventions must be framed as worthy public investments, not expensive bailouts.[9]

## Applying the four principles in phase 2

Most of the public aid provided to firms so far has been in the form of debt (loans and guarantees) and does not address solvency worries that will get worse as the crisis lengthens. At the microeconomic level, phase 2 is characterised by the need for solvency support: direct capital injections into hard-hit balance sheets.[10]

In this context, the principles discussed in the previous section suggest a large European equity fund should be created to ensure a single approach to recapitalisation measures and to protect the integrity of the single market. Indeed, the centralisation of funds would allow for a proportionate allocation and a consistent approach to helping firms in different EU countries, thus limiting distortion.

The Council 'Next Generation EU' agreement of 21 July 2020 contains one measure in this direction: a budget boost to the existing InvestEU (previously known as the Juncker Plan). InvestEU is the EU's investment fund; it mobilises public and private investment through an EU budget-guarantee backing projects of the European Investment Bank (EIB) and others.

While we welcome this proposed boost to InvestEU, this measure will be of no use in Phase 2: none of the meager €5.6 billion dedicated to the programme are allocated to 2020, when the funds are most urgently needed (Darvas, 2020b). Even past Phase 2, it will be too little. What can €5.6 billion spread over seven years achieve in the face of the estimated €350 billion to €500 billion liquidity shortfall this year (European Commission, 2020b)? And can €5.6 billion spread over the entire EU realistically level the playing field when Germany has earmarked €100 billion for recapitalisation interventions?[11] In fact, a great disappointment with the Council agreement is its abandoning of a €26 billion EU equity fund, as had been proposed by the European Commission in its Next Generation EU proposal.[12]

The details of InvestEU can still change.[13] In addition to a significantly larger budget overall and a positive budget for 2020, we recommend that the fund allocate capital ac-

7 Knowledge that a rival is barred from aggressive pricing could be an open invitation for tacit collusion.

8 None of the rescue packages given so far to airlines have included binding green conditions. See: https://www.greenpeace.org/eu-unit/issues/climate-energy/2725/airline-bailout-tracker/.

9 Lonergan and Blyth (2020) argue that 'bailout' is a misnomer in the case of COVID-19.

10 On 9 April 2020, the European Commission proposed to further extend the scope of the Temporary Framework to include direct recapitalisation measures, e.g. in the form of equity stakes and subordinated debt. See https:// ec.europa.eu/commission/presscorner/detail/en/ STATEMENT_20_610.

11 As of 10 June 2020, only €6 billion were used (direct recapitalisation through the Economic Stabilisation Fund, *Wirtschaftsstablisierungsfonds*.

12 The European Commission's 27 May 2020 proposal contained a €26 billion 'Solvency Support Instrument' to invest in coronavirus-hit businesses across Europe.

13 The Recovery Fund still needs ratification by the European Parliament.

cording to the four principles set out in the previous section.

As regards our recommended focus on viable firms, the involvement of the EIB is one of the strong points of the proposed measure. The Bank's deep ties with local partners, such as national promotional banks (e.g. ICO in Spain) and private financial institutions, mean a proven ability to leverage local knowledge. Within an EU framework, the expertise of these institutions would help direct funds towards the firms most likely to be viable in the long run.

Furthermore, conditions should be attached to the disbursed funds, ensuring accelerated changes towards agreed common societal goals. Better still, the fund should be managed for the public's benefit, and the profits dedicated to financing societal goals at the local level, thus providing a clear social sharing of the upsides. European taxpayers would thus not be bailing out firms, but would rather be investing in them. If well designed, this would not lead to systematic cross-border transfers because equity support would be given on the condition of receiving a share of future profits.

In terms of instruments, equity or equity-type instruments (e.g. transfers with a remuneration contingent on future profits[14]) are preferable to pure transfers or subordinated debt instruments because they allow for a share in future profits. However, care should be taken to limit the distortionary effects of pure equity instruments. Equity should be (i) without voting rights, (ii) with quantitative limits, (iii) with a timeline for government exit. For SMEs, equity-type instruments may be preferred to pure equity because of the known problems associated with valuing equity stakes in closely held SMEs. For example, Boot et al. (2020) propose injecting cash in exchange for future higher tax payments (conditional on the firm having recovered).

Short of a pan-European fund, the most effective way to limit the distortionary effects of state subsidies is crude and mechanical: state-aid exemptions must be short-lived and enforcement must be biting. This would risk too little state support, without common societal goals.

## Principles in phase 3: Towards a strong and sustainable recovery

Even if a COVID-19 vaccine becomes available, it will likely take several years until the level of economic activity of 2019 will be reached, for three reasons.

First, despite all the government support provided, many firms will have disappeared. Valuable physical, financial and human capital will have been lost. Rebuilding new productive structures will take time and investment.

Second, households have suffered a major shock to their incomes and have reduced savings. They will want to rebuild their savings as soon as incomes recover. It is therefore entirely possible that the private savings rate will be higher post-lockdown, putting a drag on demand.

Third, global value chains could be significantly disturbed for some time because of the different stages of the virus and vaccination, and because of private and public responses to the experience. This could reduce productivity.

In phase 3, the EU must play a major role – through the MFF and the new recovery facility, 'Next Generation EU' – alongside national recovery programmes. As phases 2 and 3 are intrinsically linked, measures should be based on the same objectives. In light of that, we discuss the key principles of a recovery initiative/fund.

Next Generation EU responds to the need to counterbalance the huge differences between countries' fiscal room to manoeuvre and abilities to boost their economies. Notably, it aims at preventing two scenarios.

First is a rise in spreads that would render debt unsustainable and self-fulfilling crises more likely. Indeed, by relying exclusively on national borrowing, the debt of some countries could become difficult to fund on primary (and even secondary) markets. Second is underinvestment. Fearing market reactions, countries may borrow too little, thereby supporting their economies insufficiently and doing long-term damage to both EU economic performance and political cohesion.

Under the European Council's July 2020 agreement, Next Generation EU would be financed by long-term EU borrowing. As such, it represents significant cross-country insurance. Disbursed mostly in the form of grants, followed by loans and guarantees, the facility would support countries' primary markets (by avoiding an extra budgetary burden) and debt sustainability (with grants and by passing on the interest rate advantage of EU debt).

Next Generation EU will thus be crucial in the recovery phase. However, its design should be based on four guidelines.

*First, the recovery fund needs to focus on broader EU societal goals*. The EU has committed to lead the transition to a healthier planet and a new digital world (von der Ley-

14 See the SAFE proposal: https://voxeu.org/article/ implementing-europeanpandemic-equity-fund.

en, 2019), and it is important that both demand and supply support measures promoted under the Next Generation EU be consistent with these broader societal goals. To that effect, the twofold increase in the budget of the Just Transition Fund – the EU programme that provides assistance to EU territories most negatively affected by the transition to a climate-neutral economy – is a welcome feature of the recovery facility (though it may not be enough, see Cameron et al. 2020).[15] So is the stipulation that only countries that commit to climate neutrality by 2050 be eligible for full funding.

*Second, the recovery fund needs to be financed primarily through borrowed money*. It is optimal to smooth the consequences of a large shock over time, i.e. through borrowing. Wolff (2020) argued that EU borrowing is the way forward to fund the costs currently being incurred. In the monetary union in particular, such EU borrowing would bring significant advantages and strengthen the euro area macroeconomy, while helping to overcome the problems of single market fragmentation that result from primarily national responses. As discussed above, purely national borrowing would weaken the single market and also render the monetary union more fragile. We thus welcome Next Generation EU's reliance on long-term EU borrowing.

*Third, the recovery fund needs to strike the right balance between grants, loans and accountability*. Traditional European Commission schemes, from the Juncker Plan to InvestEU, up to the recently proposed European Green Deal Investment Plan, tend to focus on financial architecture based on guarantees and loans, in order to trigger large-scale private and public investment initiatives. Such initiatives have been received sceptically in the past, given the uncertainties about their real additionality (Claeys and Leandro, 2016; Claeys and Tagliapietra, 2020).

Given the unprecedented uncertainty faced by companies in the COVID-19 crisis, it is important that a large share of the recovery come in the form of grants. The current €750 billion agreement includes €390 billion in grants. However, a mere 25% would likely be spent in 2020-2022, when the recovery needs will be greatest (Darvas, 2020a).[16]

While grants provide more insurance, they also imply bigger transfers and are more politically charged. Their legitimacy and accountability is more difficult to establish. Ultimately, a system with large amounts of European grants requires a European spending programme with central control, accountability and enforcement. Indeed, providing grants centrally while exercising spending decisions nationally is incompatible with legitimacy and accountability.

*Fourth, the EU budget's structure and allocation methods should be rethought*. President von der Leyen claimed she can turn the EU's budget into the "mothership" of the European recovery (European Commission, 2020). But in order to deliver on the objective of an effective economic recovery aligned with broader societal goals, the EU budget needs a structural rethink.

The 2014-2020 EU budget was predominantly focused on the Common Agricultural Policy (CAP) and Structural and Cohesion Funds (together making up 71% of spending; Moes, 2018). The economic literature shows that the CAP provides good income support, especially for richer farmers, but is less effective for greening and biodiversity and is unevenly distributed. The literature also shows great uncertainty over the real size and effectiveness of cohesion policy (Darvas and Wolff, 2018). In the wake of COVID-19, the EU budget should be targeted more at the sectors of the future – such as green and digital – and made more efficient and effective. It is thus disappointing that the Council's agreement misses the opportunity for a fundamental reform of the EU budget, including the CAP.

Finally, the way resources are allocated really matters. A significant part of spending should be targeted at the European regions most affected by COVID-19.[17] To do so, it will be essential to introduce into the allocation methods a set of parameters that prioritise regions that have been impacted most by COVID-19, in both health and economic terms. The European Council's agreed recovery instruments, however, only partially fulfil this requirement. Significant parts of the overall programme are allocated exclusively on the basis of 2019 and prior data, and not on measures of the size of shock. As regards the main instrument for example (the €672.5 billion Recovery and Resilience Facility), it is only the allocation for the year 2023 that will account for GDP loss caused by the crisis.[18] This may be too late – and too little given that most of the instrument is (rightly) allocated to 2021-2022. Targeting the programme more specifically to those regions most affected by the economic recession in the coming years is a worthwhile discussion going forward.

15 Note, however, that the Council agreement more than halved the Just Transition Fund's budget compared to the European Commission's 27 May 2020 proposal, from €40 billion to €17.5 billion.

16 Provided the recovery instrument is subjected to the same time constraints as usual, which was the case under the 27 May proposal.

17 Wolff (2020) discussed whether and to what extent this creates moral hazard concerns.

18 Real observed GDP loss over 2020 and cumulative loss in real GDP observed over the period 2020-2021.
